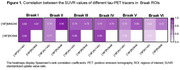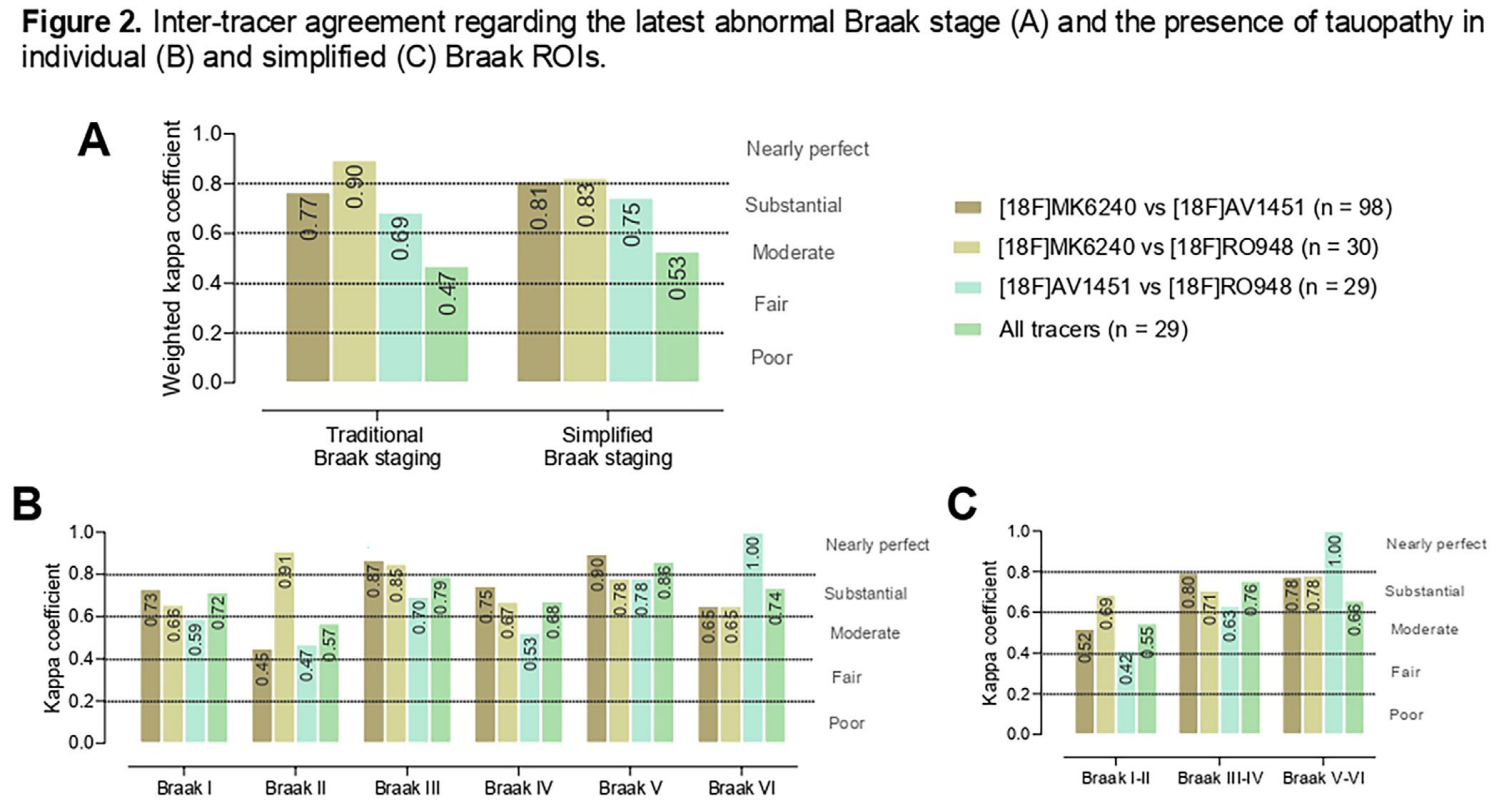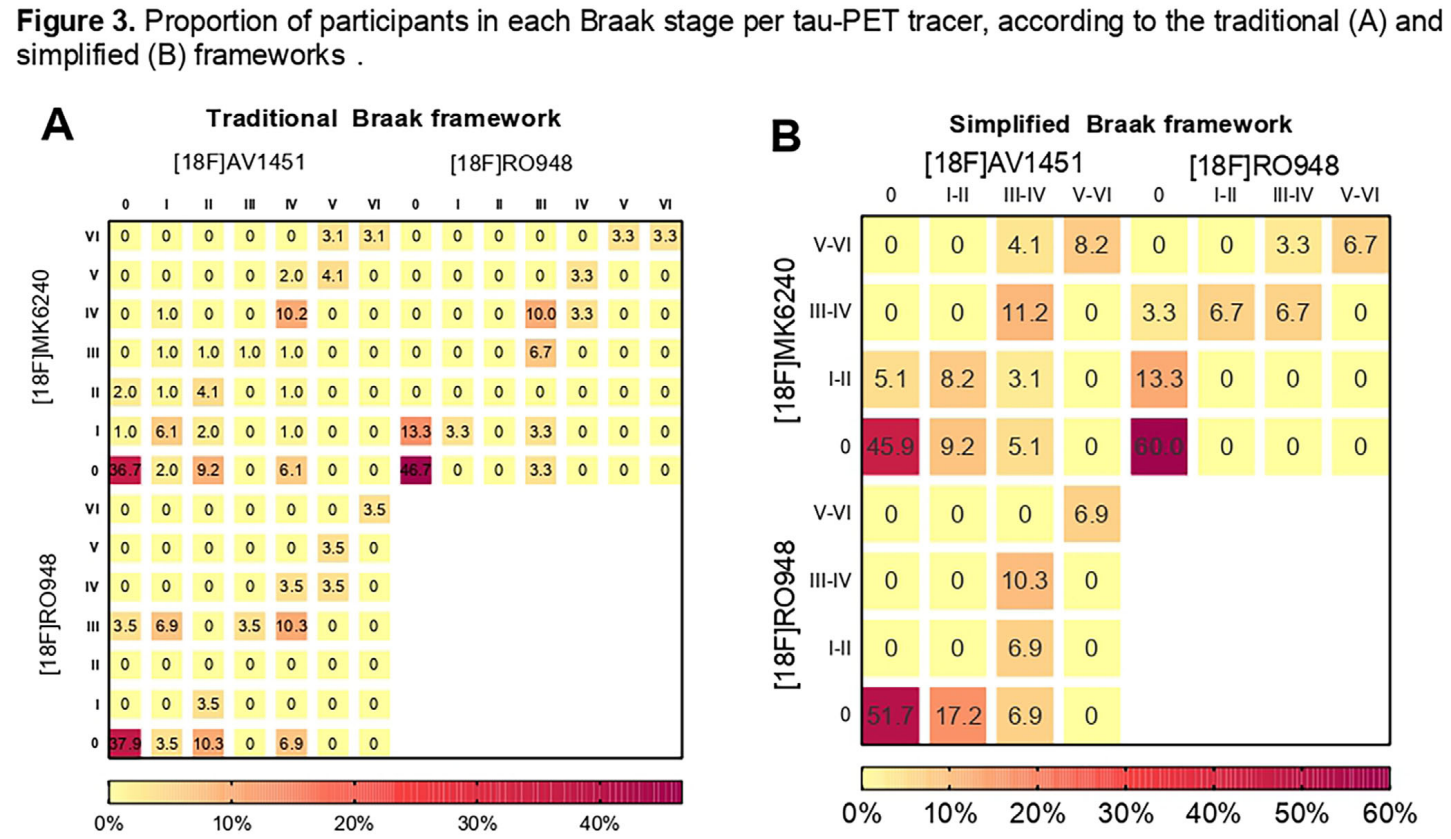# Comparison of tau‐PET tracers for in vivo Braak staging: the HEAD Study

**DOI:** 10.1002/alz.093737

**Published:** 2025-01-09

**Authors:** Arthur C. Macedo, Joseph Therriault, Nesrine Rahmouni, Stijn Servaes, Yi‐Ting Wang, Cécile Tissot, Firoza Z Lussier, Jaime Fernandez Arias, Kely Monica Quispialaya Socualaya, Seyyed Ali Hosseini, Pamela C.L. Ferreira, Bruna Bellaver, João Pedro Ferrari‐Souza, Cristiano Schaffer Aguzzoli, Guilherme Povala, Belen Pascual, Brian A. Gordon, Val J. Lowe, Hwamee Oh, David N. Soleimani‐Meigooni, Suzanne L. Baker, Tharick Ali Pascoal, Pedro Rosa‐Neto

**Affiliations:** ^1^ McGill University, Montreal, QC Canada; ^2^ University of Pittsburgh, Pittsburgh, PA USA; ^3^ Houston Methodist Research Institute, Houston, TX USA; ^4^ Washington University in St. Louis, Saint Louis, MO USA; ^5^ Department of Radiology, Mayo Clinic, Rochester, MN USA; ^6^ Brown University, Providence, RI USA; ^7^ Memory and Aging Center, Weill Institute for Neurosciences, University of California, San Francisco, San Francisco, CA USA; ^8^ Lawrence Berkeley National Laboratory, Berkeley, CA USA

## Abstract

**Background:**

Tau‐PET tracers allow for in vivo Braak staging of individuals in the Alzheimer’s disease (AD) continuum. The impact of tracers’ characteristics for Braak staging using tau‐PET remains unclear. Therefore, we performed a head‐to‐head comparison of Braak staging using first‐ and second‐generation tau‐PET tracers.

**Method:**

We assessed 51 cognitively unimpaired (CU) and 49 cognitively impaired participants (mean [SD] age 69.4 [7.5] years) with at least two tau‐PET ligands ([18F]MK6240, [18F]AV1451, and/or [18F]RO948) at McGill University, as part of the HEAD study. We calculated standardized uptake value ratios (SUVR) in Braak‐like regions of interest (ROI) for each ligand and investigated their association using Spearman’s correlation. Thresholds defined the presence of tauopathy in each Braak ROI, which was used to assign a Braak stage to each participant. Finally, we assessed the agreement between the Braak staging provided by each tracer, both for the traditional (using separate Braak stages – 0, I, II, III, IV, V, and VI) and simplified (using joint Braak stages – 0, I‐II, III‐IV, and V‐VI) frameworks.

**Result:**

In all Braak ROIs, we found positive correlations between the SUVR values of the three tracers (Fig. 1). The strongest correlation was between [18F]MK6240 and [18F]RO948 in Braak II (r=0.94), and the weakest between [18F]AV1451 and [18F]RO948 in VI (r=0.51). Inter‐tracer agreement regarding the latest stage of abnormality was substantial or nearly perfect between pairs of tracers but moderate between the three tracers (Fig. 2A). The simplified framework presented greater agreement compared to traditional Braak staging, except between [18F]MK6240 and [18F]RO948. At the ROI level, the lowest agreements were observed for Braak II and I‐II, when [18F]MK6240 and [18F]RO948 were compared to [18F]AV1451 (Fig. 2B‐C). [18F]AV1451 had the highest probability of overestimating the Braak stage assigned by [18F]MK6240 and by [18F]RO948, especially at earlier stages (Fig. 3).

**Conclusion:**

The ligands presented moderate to nearly perfect agreement for in vivo staging of AD severity. Off‐target binding of [18F]AV1451 to the choroid plexus might explain disagreements at early stages. Overall, slight improvements in agreements were achieved with the simplified framework, as observed in histopathological staging.